# Supplementary material for: MYC promotes immune-suppression in triple-negative breast cancer via inhibition of interferon signaling
Source: Nat Commun. 2022 Nov 2;13:6579. doi: 10.1038/s41467-022-34000-6 (PMC9630413; doi:10.1038/s41467-022-34000-6)
Supplement: Supplementary file 1 — Supplementary Information [file 41467_2022_34000_MOESM1_ESM.pdf]

Supplementary information file

MYC promotes immune-suppression in triple-negative breast cancer via inhibition of interferon signaling

Supplementary Figure S1

A

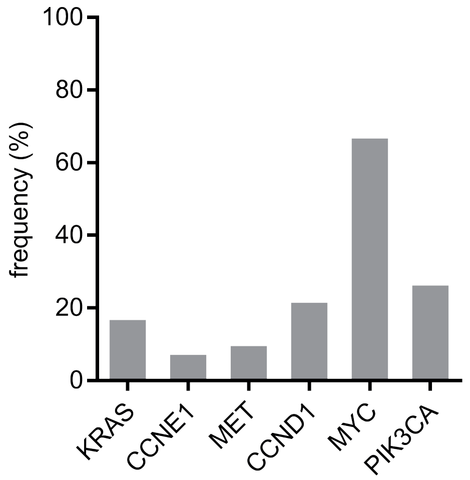

B

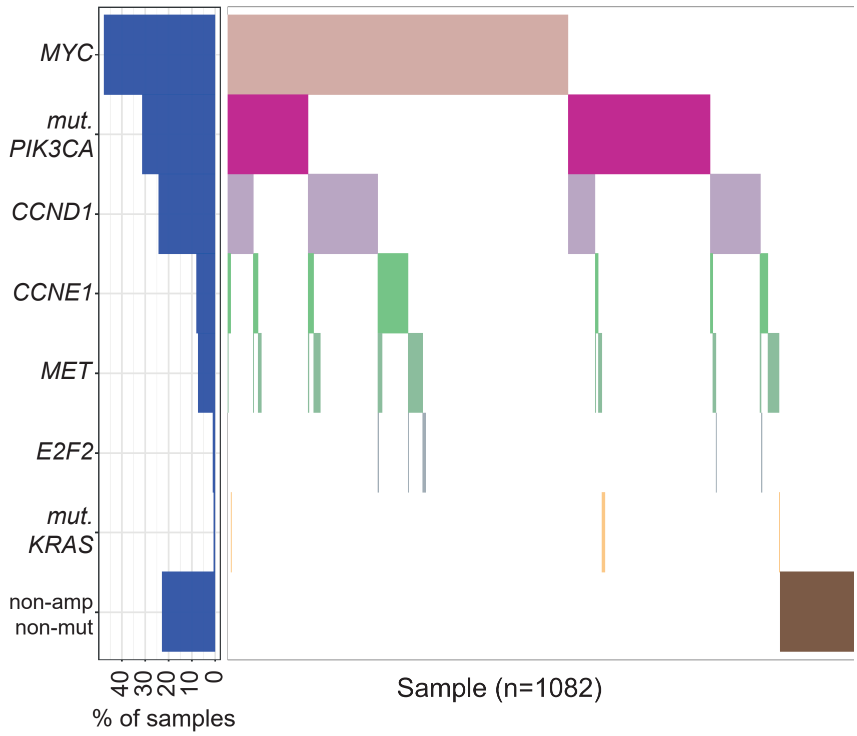

C

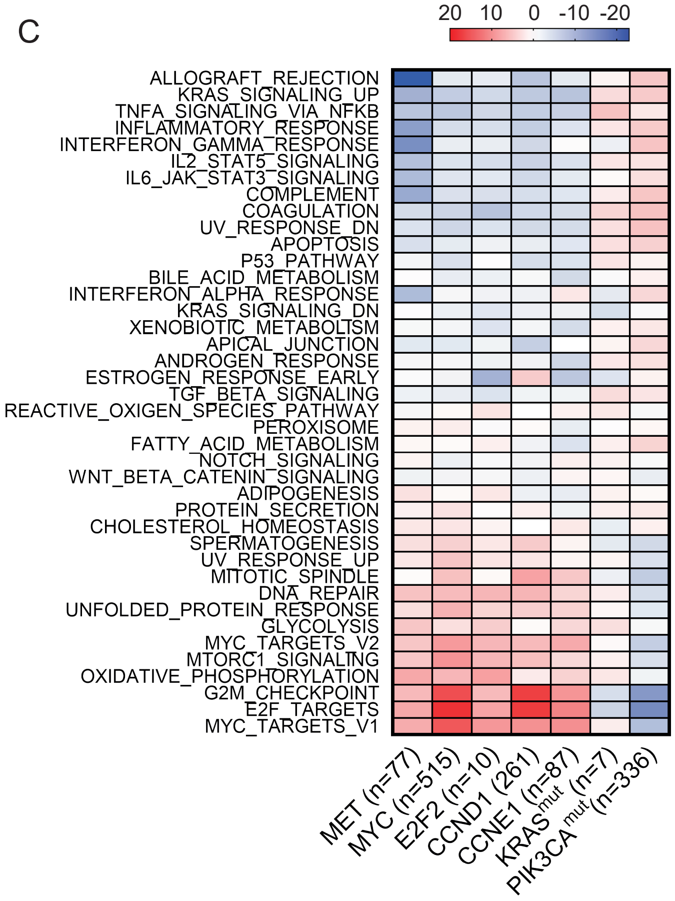

Supplementary Figure S1 – Oncogene amplifications in human breast cancer

Related to Figure 1.

(A) Frequency of amplified oncogenes in known *BRCA1/2*-mutated breast cancer samples (n=42) from TCGA data.

(B) Distribution plot of all breast cancer samples used for GSEA analysis from TCGA data. In total, 1028 breast cancer samples were included in the analyses. Individual samples are plotted on the x-axis, oncogenic aberrations on the y-axis.

(C) GSEA analysis of breast cancer samples (n=1082). Top 20 up (red) and down (blue) regulated hallmark gene sets are plotted in amplified vs neutral samples for specific oncogenes. Number of amplified samples per oncogene are shown. Values plotted are Z-transformed p values.

Supplementary Figure S2

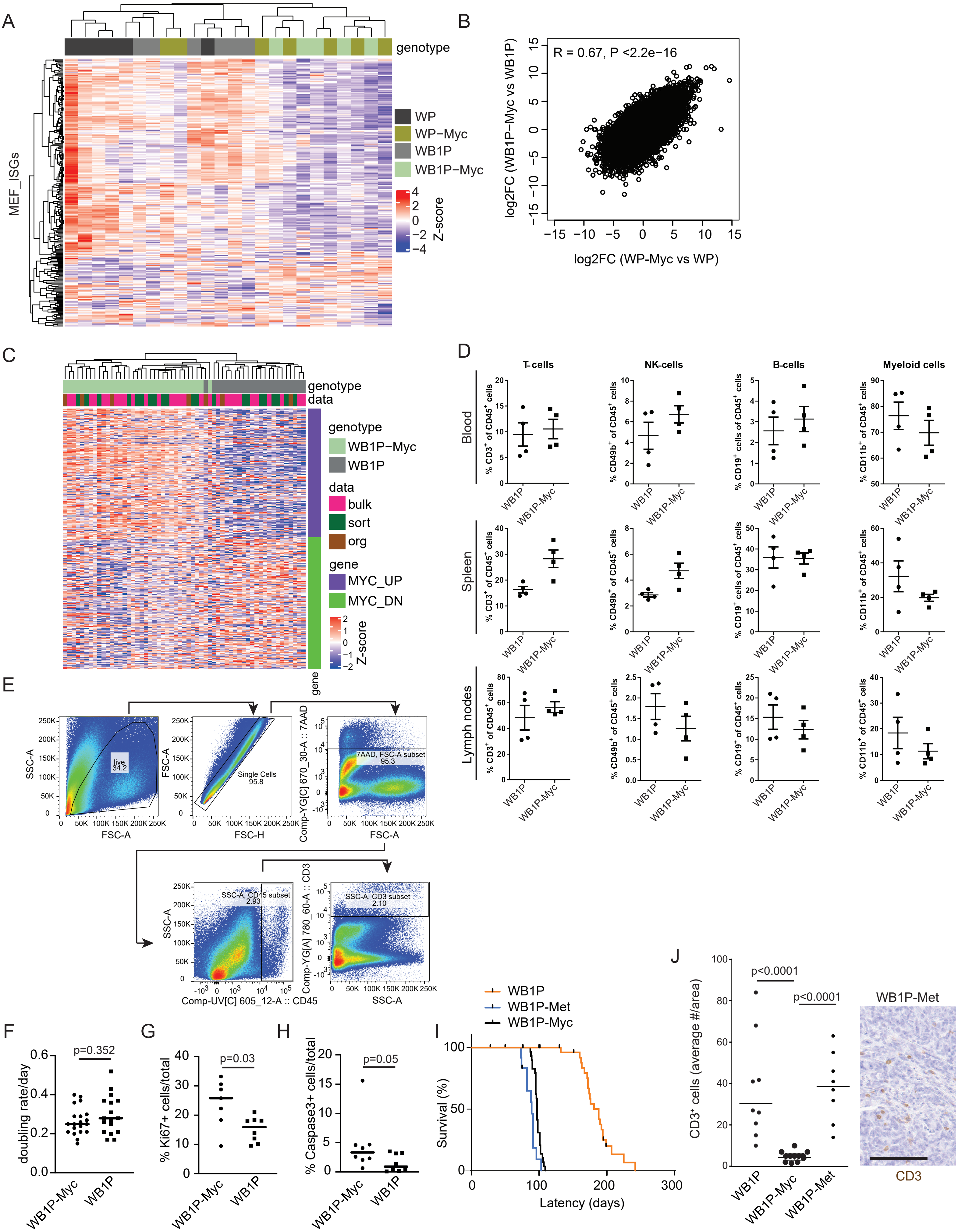

Supplementary Figure S2 – MYC activation alters tumor immunity in TNBC mouse models

Related to Figure 2.

(A) Heatmap for the previously reported interferon-stimulated genes (Mackenzie et al., 2017) in RNA-seq of WB1P, WB1P-Myc, WP, and WP-Myc models.

(B) Scatter plot of the correlation of expression changes between the WB1P and WP tumor models upon MYC overexpression. The correlation between MYC expressing tumors is independent of BRCA1 status, (P values were calculated with two-tailed t-transformations of Pearson's R correlation coefficients, $p<2.2e-16$, Pearson correlation coefficient $R=0.67$).

(C) Heatmap for the previously reported MYC signatures (Bild et al., 2006) in RNA-seq of WB1P-Myc and WB1P bulk tumor, sorted tumor cells and organoids.

(D) Flow cytometry analysis of $CD3^{+}$, $CD49b^{+}$, $CD19^{+}$, $CD11b^{+}$ cell presence in blood, spleen and lymph nodes of WB1P and WB1P-Myc mice showing no pronounced changes in peripheral immunity in the WB1P-Myc mice (n=4, mean with SEM plotted).

(E) Gating strategy of the FACS experiments shown in supplementary Figure S2D and in Figure 2D.

(F) Quantification of the doubling rate/day of WB1P-Myc (n=20) and WB1P (n=17) tumors. Doubling rates/day were calculated by using the formula: doubling time (DT)=0.69/r (rule of 69), from this follows the doubling rate of 1/DT. (unpaired two-tailed Mann-Whitney test, $p=0.352$, each dot represents an independent tumor, mean with SD plotted).

(G) Quantification of proliferative cells in WB1P-Myc and WB1P tumors. Sections of 8 WB1P and 6 WB1P-Myc tumors were stained with an antibody against Ki67 and the percentage of positive cells on the whole slide was counted with quPath. (unpaired two tailed Mann-Whitney test, $p<0.05$, each dot represents an independent tumor, mean with SD plotted).

(H) Quantification of apoptotic cells in WB1P-Myc and WB1P tumors. Sections of 8 WB1P and 8 WB1P-Myc tumors were stained with an antibody against cleaved caspase-3 and the percentage of positive cells in the whole section was counted with quPath (unpaired two tailed Mann-Whitney test, $p<0.05$, each dot represents an independent tumor, mean with SD plotted).

(I) Latency analysis of WB1P vs WB1P-Met vs WB1P-Myc tumors.

(J) Representative histogram of CD3 immunostaining in WB1P-Met tumor. $CD3^+$ cell counts are quantified for WB1P (n=9) vs WB1P-Myc (n=12) vs WB1P-Met (n=8) tumors (see also Figure 2C), two tailed unpaired Mann-Whitney test, $p<0.0001$, each dot represents an independent tumor, mean with SD plotted, scale bar = 100μm.

Supplementary Figure S3

A

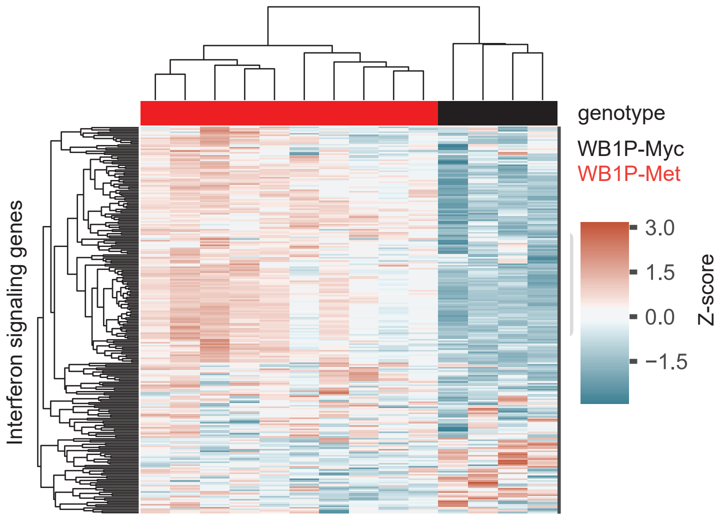

B

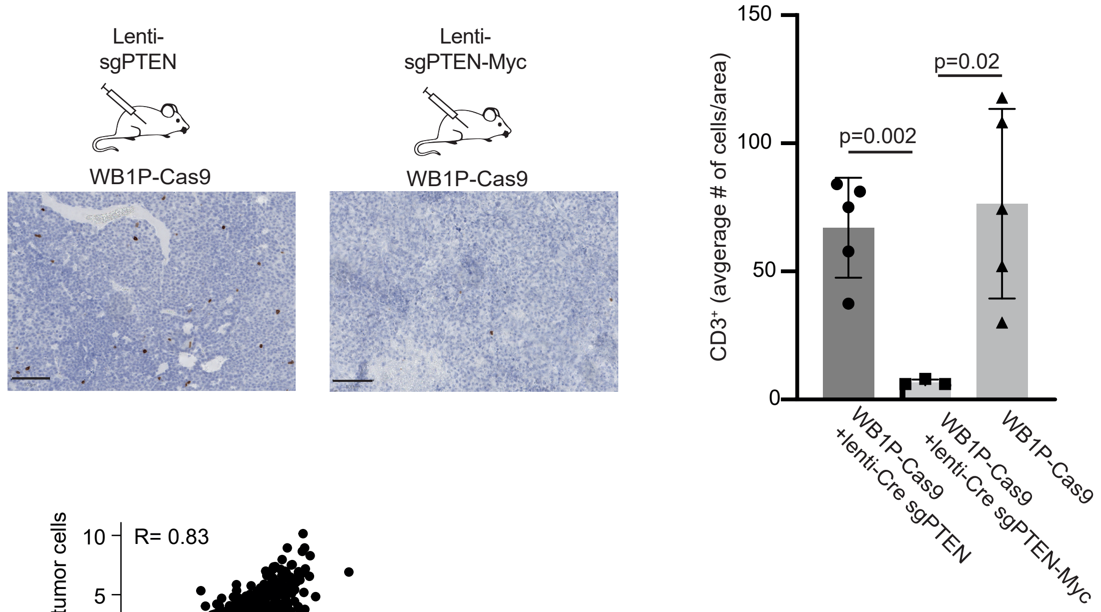

C

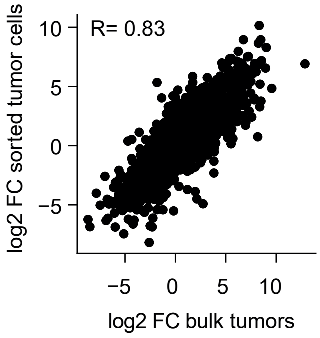

D

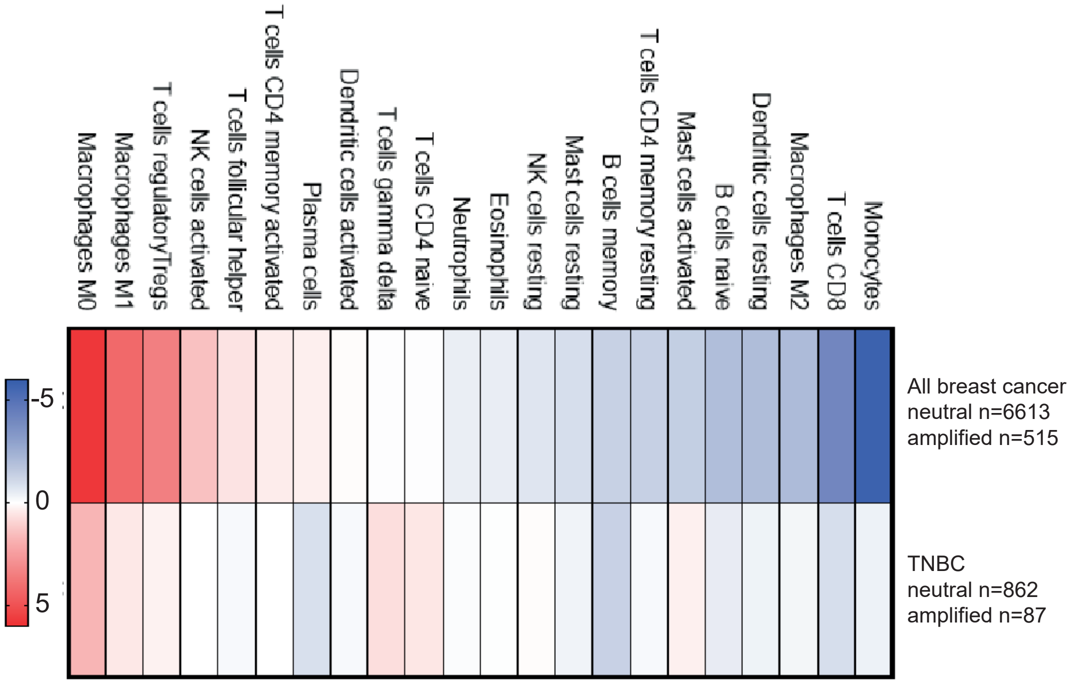

Supplementary Figure S3 – The shorter latency until tumor onset is not causal for the reduced immune infiltration in MYC overexpressing tumors

Related to Figure 2.

(A) Heatmap of RNA-seq of WB1P-Myc vs WB1P-Met tumors. An IFN signature of 336 genes was used (Saleiro et al., 2015), showing the immunogenicity of WB1P-Met tumors.

(B) Representative images of $CD3^+$ staining for tumors induced by intraductal injections of guides against PTEN with (n=3) and without MYC overexpression (n=5) and a WB1P-Cas9 control (n=5). Right panels: Counts of $CD3^+$ cells in defined areas (two tailed student`s t-test, $p<0.05$; scale bar=100µm, mean with SD plotted).

(C) Scatter plot showing the correlation of expression changes between WB1P bulk tumors and sorted epithelial tumor cells by overexpression of MYC (R=0.83).

(D) Distribution of immune cell–type fractions in all breast cancer samples and TNBC only from TCGA were estimated with CIBERSORT analysis. Samples with amplified MYC (0.3 cut-off) were compared to samples with neutral MYC levels. Fractions of each immune cell type were compared with a Welch's t-test. -log10(pvalue)*sign(t statistic) for each immune cell type are plotted. Color indicates a lower (blue) or higher (red) immune cell-type fraction in breast cancer samples with amplified MYC compared to neutral MYC samples.

Supplementary Figure S4

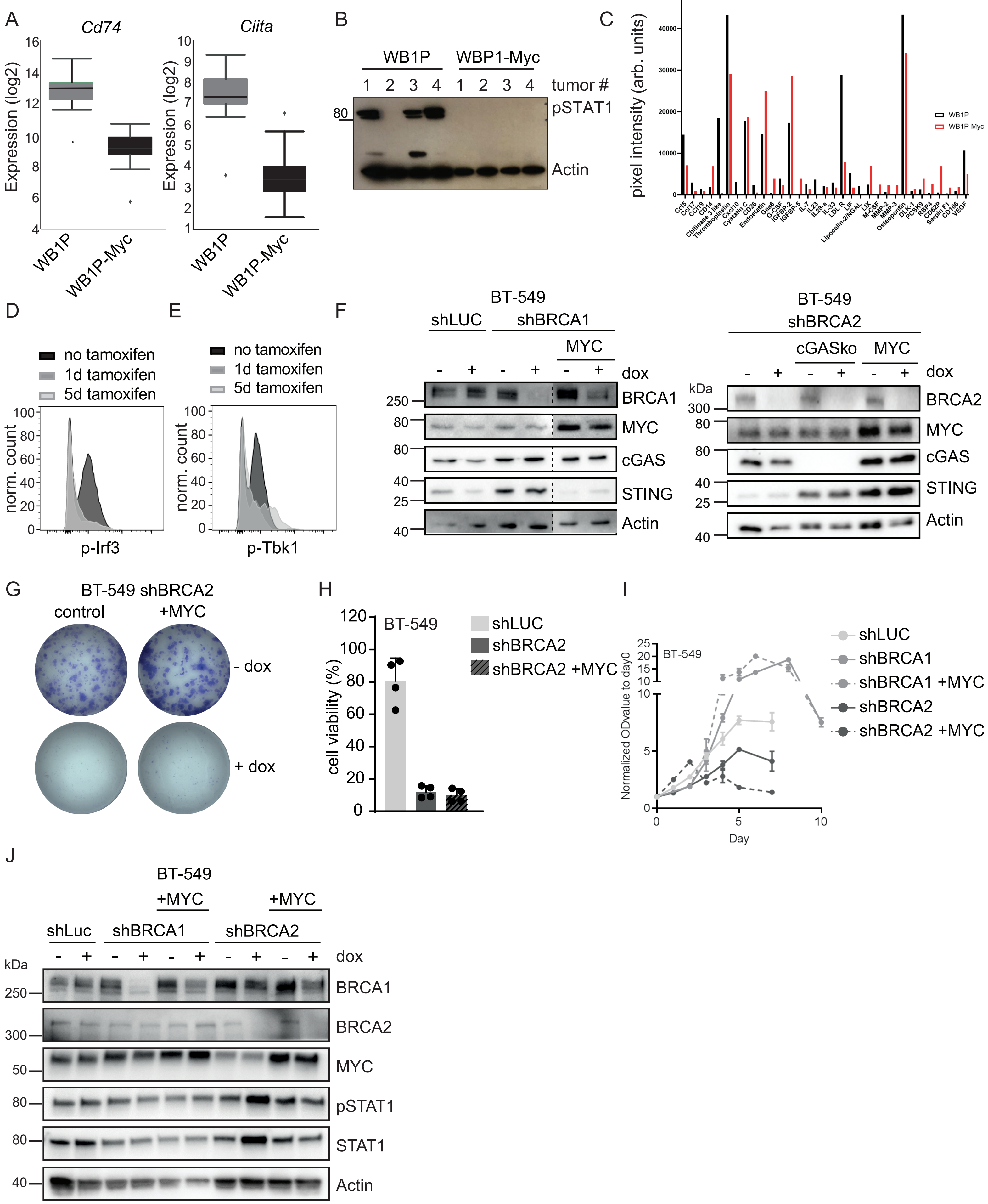

Supplementary Figure S4 – cGas/STING and IFN signaling is suppressed by MYC in mouse and human organoid and cell line models

Related to Figure 3.

(A) Boxplots from RNA-seq data showing downregulation of CD74 and CIITA in WB1P (n=11) and WB1P-Myc (n=18) tumors. Boxplots are represented as median (centre line) ± IQR (25th to 75th percentile, box) and IQR ± 1.5 x IQR (whiskers).

(B) Western blot showing the reduced pSTAT1 expression in WB1P-Myc tumors, of 4 independent tumors/genotype.

(C) Cytokine array on supernatants of WB1P and WB1P-Myc organoids. Pixel intensity of spots was measured via ImageJ after background subtraction (data from one array shown, array performed in duplicate).

(D) Representative image of FACS of p-IRF3 expression in WB1P-Myc^{ERT2} organoids with 1 day tamoxifen (dark grey), 5 days tamoxifen (light grey) and without (black), (see also Figure 3E).

(E) Flow cytometry analysis of p-TBK1 in WB1P-Myc^{ERT2} tumor epithelial organoids without tamoxifen (black), after 1 day with tamoxifen (dark grey) and after 5 days of tamoxifen treatment (light grey).

(F) BT-549 and BT-549-cGAS$^{-/-}$ cells with indicated hairpins were depleted for BRCA1 or BRCA2, or overexpressed for MYC. Cells were treated with or without dox for three days, and immunoblotted for BRCA1, BRCA2, MYC, cGAS, STING and Actin.

(G) Representative image of long-term survival assay in BT-549. BT-549 cells harboring shBRCA2 with or without WZL-MYC were plated in 6-well plates and treated with or without dox. Cells were fixed after 10-14 days and stained with crystal violet.

(H) Quantification of long-term survival assay as described in G. BT-549 cells were plated in 6 wells with indicated hairpins with or without MYC overexpression and treated with dox. Percentage of cell survival was calculated by normalizing measurements to wells without dox treatment. Data of 4 biological replicates are plotted. Averages and error bars (SD) are indicated.

(I) Cell proliferation of BT-549 cells was analyzed with SRB assays. Equal numbers of cells were plated in 48 wells plates and treated with or without dox. At indicated time points, cells

were fixed and stained with SRB dye. OD values of dissolved SRB dye were normalized to day 0 of the same cell line. Three biological replicates are plotted. Averages are indicated, along with error bars that reflect standard deviation.

(J) Control or MYC overexpressing BT-549 cells harboring shBRCA1/2 were treated with doxycycline (dox) for three days, and were immunoblotted with indicated antibodies (representative image of 3 independent experiments shown).

Supplementary Figure S5

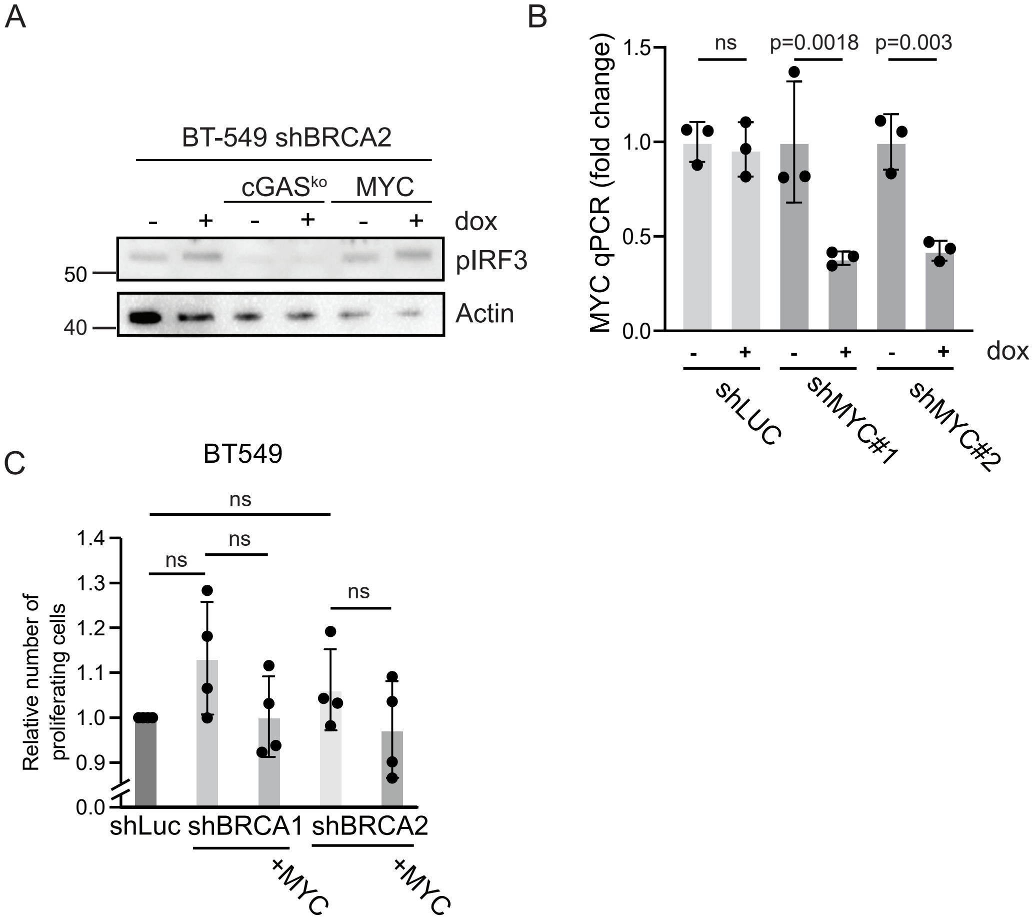

Supplementary Figure S5 – IFN signaling in TNBC human cell lines is suppressed by MYC

Related to Figure 3 and 4.

(A) shBRCA2-BT-549 cells, wt or mutant for cGAS or with MYC overexpression were treated with or without doxycycline for 5 days. Phosphorylation status of IRF3 was analyzed by immunoblotting.

(B) BT-549 cells were stably transduced with doxycycline (dox)-inducible shRNAs targeting *MYC* or *LUC,* and were cultured for three days with dox. q-RT-PCRs for *MYC* were performed to analyze efficiency of MYC depletion. Data were normalized to cell lines without dox treatment. Averages and standard deviation are plotted of n=3 biological replicates. P-values were calculated using one-way ANOVA with Sidak's multiple comparisons test.

(C) The activation of T cells was measured by the dilution of the 'violet celltrace' marker per cell division. The percentage of activated CD8+ T cells was measured by flow cytometry 5 days after co-culture with harvested medium from BT-549 cells pre-treated with doxycycline. Averages are indicated with error bars reflecting standard deviations of n=4 independent experiments. Two-tailed student`s t-tests, all non-significant. P-values were calculated using one-way ANOVA with Sidak's multiple comparisons test.

Supplementary Figure S6

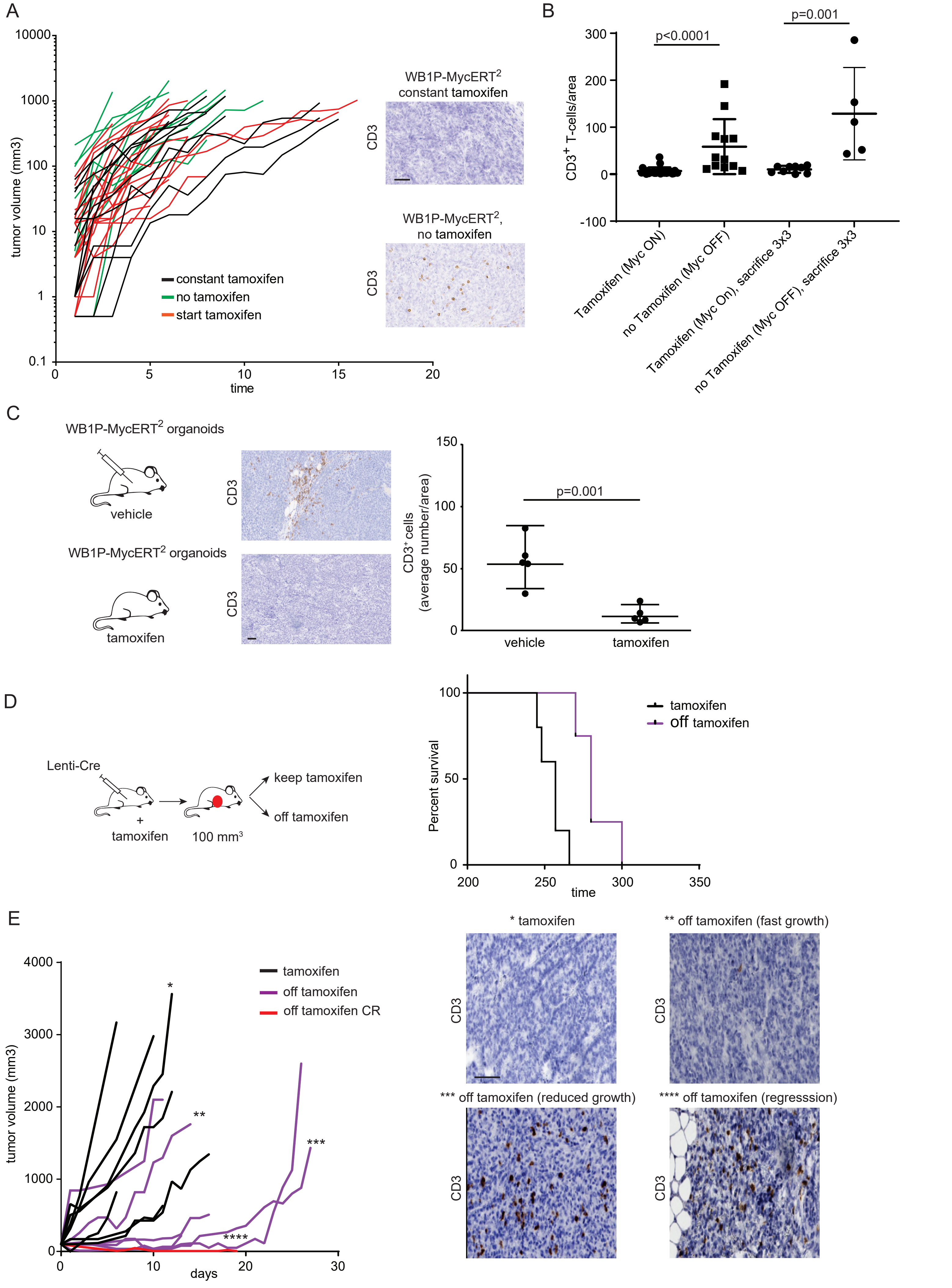

Supplementary Figure S6 – Inducing MYC in existing tumors leads to expulsion of immune infiltrates

Related to Figure 4

(A) Growth curves of WB1P-Myc^{ERT2} tumors with (black), without (green) and with tamoxifen from tumor size of 3x3 mm (red). On the right side are representative micrographs of CD3 IHC in tumors with and without tamoxifen (scale bar=50µm).

(B) Assessment of tumor infiltrating CD3^{+} T cells in tumors harvested at a size of 15x15mm respectively 3x3mm with (n=32 for 15x15mm and 9 for 3x3mm) and without Tamoxifen (n=12 for 15x15mm and 5 for 3x3mm) induced MYC activation (every dot represents an individual tumor, unpaired two sided Mann-Whitney test, $p<0.0001$ for 15x15mm and $p=0.001$ for 3x3mm, mean with SD plotted).

(C) WB1P organoids with a MYC^{ERT2} vector are orthotopically transplanted into mammary glands. Transplanted mice are treated with tamoxifen chow and resulting tumors assessed for CD3^{+} T cell infiltration (IHC, CD3 in brown), quantification in right panel, n=5 mice/group, 5 windows/tumor counted, unpaired two tailed t-test, $p=0.001$, scale bar = 50µm, mean with SD plotted).

(D) Kaplan-Meyer curves of Myc^{ERT2}-P2A-Cre injected B1P mice with tamoxifen and after tamoxifen withdrawal (scale bar = 50µm).

(E) Relative tumor growth (left) of intraductally injected WB1P mice with Lenti-Cre MYC^{ERT2}. Tamoxifen administration and immunostaining for CD3^{+} (right), in selected mice with (*) showing that concomitant MYC de-activation and slower tumor progression is paired with increase of infiltrating lymphocytes and complete regression (CR) in one of the mice (scale bar = 50µm).

Supplementary Figure S7

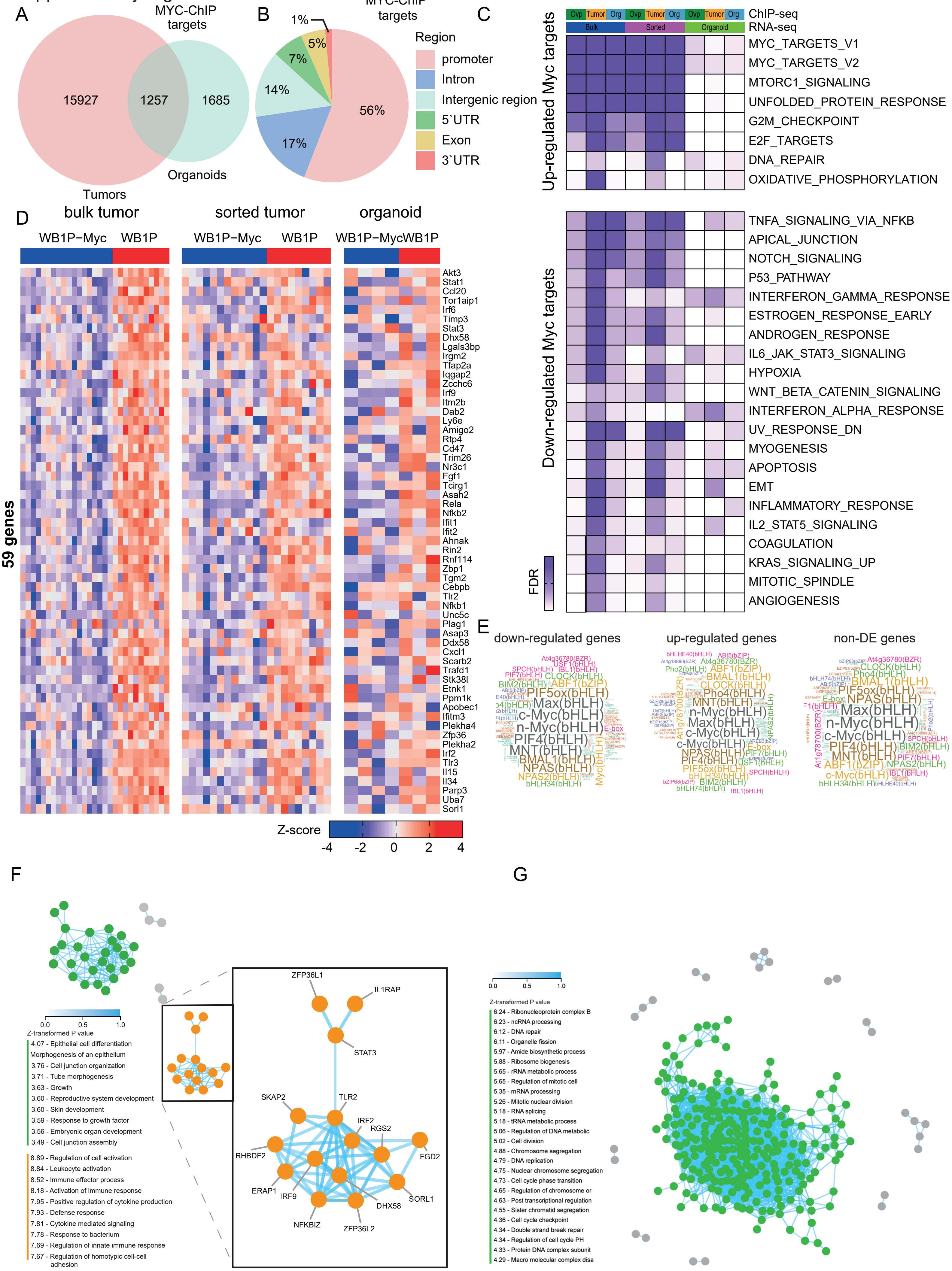

Supplementary Figure S7 – Integration of MYC ChIP-seq and RNA-seq of tumors and organoids shows MYC mediated direct inhibition of immunomodulatory factors downstream and in parallel of c-GAS/STING signaling

Related to Figure 5

(A) Overlap of the MYC-binding loci obtained from the tumor and organoid ChIP-seq data.

(B) Genomic distribution of the common MYC-binding loci between tumor and organoid ChIP-seq data.

(C) Gene ontology analysis of differentially expressed genes in WB1P vs WB1P-Myc tumors, sorted epithelial tumor cells and organoids overlapped with MYC ChIP-seq peaks.

(D) Heat maps for the 59 downregulated genes involved in interferon signaling from the RNA-seq data of bulk, sorted tumor cells and organoids with a peak called in tumor and/or organoid ChIP-seq.

(E) Transcription factor motif enrichment of the sequences bound by MYC in the ChIP-seq in up-regulated, down-regulated and not transcriptionally affected genes, confirming the MYC motif as the predominantly found motif.

(F) Left: constructed co-functionality network of genes downregulated by MYC (n=129) retrieved from both MYC-ChIP-seq of WB1P-Myc and RNA-seq data of WB1P and WB1P-Myc tumors and organoids. Right: one of the two resulting clusters in which genes share strong predicted co-functionality ($r > 0.5$) and show enrichment for immunity pathways (e.g. leukocyte activation, activation of immune response and positive regulation of cytokine secretion).

(G) Constructed co-functionality network of genes upregulated by MYC (n=430) retrieved from overlapping MYC-ChIP-seq peaks with RNA sequencing data of WB1P and WB1P-Myc tumors and organoids. Genes share strong predicted co-functionality ($r > 0.5$) within network that was enriched with genes predicted to be involved in e.g. DNA repair and RNA processing.

Supplementary Figure S8

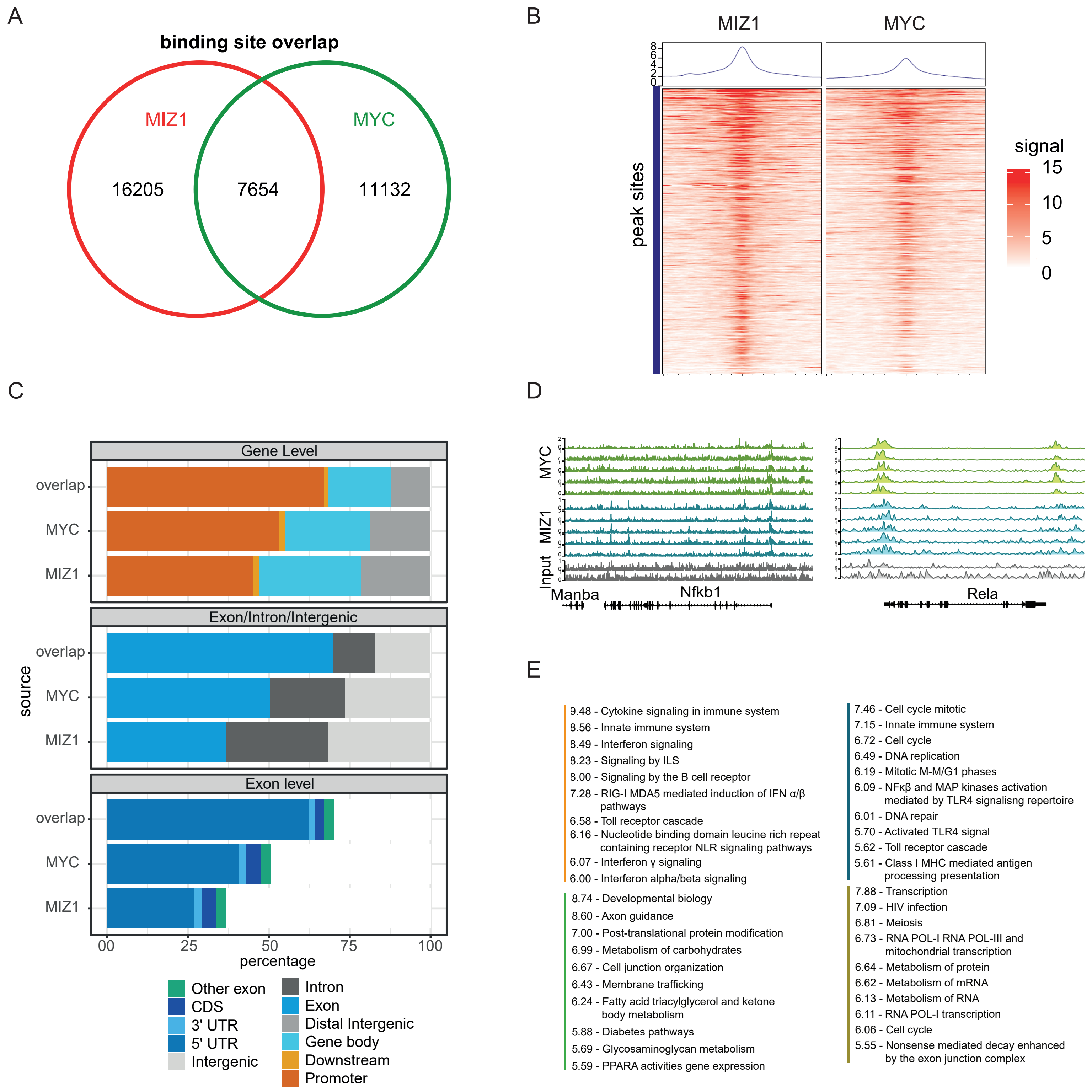

Supplementary Figure S8 – Overlay of MYC and MIZ1 ChIP-seq confirms co-occupancy of IFN-signaling promoters by MYC and its co-repressor MIZ1, suggesting direct repression of innate immunity pathways

Related to Figure 5

(A) Venn diagram showing the binding site overlap of MIZ1 with MYC ChIP-seq peaks of 5 independent WB1P-Myc tumor samples.

(B) Heat-map showing overlap of MYC and MIZ1 binding events in WB1P-Myc tumor samples.

(C) Analysis of genomic region where MYC and MIZ1 bind and overlap on the gene level, in intergenic regions and in exons.

(D) Example binding plots of MYC and MIZ1 at Nfkb1 and Rela loci.

(E) Specified lists of enriched pathways in the co-functionality network analysis of Figure 5F.
