# Supplementary material for: MYC promotes immune-suppression in triple-negative breast cancer via inhibition of interferon signaling
Source: Nat Commun. 2022 Nov 2;13:6579. doi: 10.1038/s41467-022-34000-6 (PMC9630413; doi:10.1038/s41467-022-34000-6)
Supplement: Supplementary file 3 — Description of Additional Supplementary Files [file 41467_2022_34000_MOESM3_ESM.pdf]

Description of Additional Supplementary Files

File Name: Supplementary Data 1
Description: RNA-seq results

File Name: Supplementary Data 2
Description: overlay Chip-seq and RNA-seq
