# Supplementary material for: MYC promotes immune-suppression in triple-negative breast cancer via inhibition of interferon signaling
Source: Nat Commun. 2022 Nov 2;13:6579. doi: 10.1038/s41467-022-34000-6 (PMC9630413; doi:10.1038/s41467-022-34000-6)
Supplement: Supplementary file 6 — Reporting Summary [file 41467_2022_34000_MOESM6_ESM.pdf]

nature research

Corresponding author(s): Rudolf S.N. Fehrmann, Marcel A.T.M. van Vugt, Jos Jonkers

Last updated by author(s): 11/09/2022

Reporting Summary

Nature Research wishes to improve the reproducibility of the work that we publish. This form provides structure for consistency and transparency in reporting. For further information on Nature Research policies, see our Editorial Policies and the Editorial Policy Checklist.

Statistics

For all statistical analyses, confirm that the following items are present in the figure legend, table legend, main text, or Methods section.

n/a	Confirmed	
☐	☒	The exact sample size (n) for each experimental group/condition, given as a discrete number and unit of measurement
☐	☒	A statement on whether measurements were taken from distinct samples or whether the same sample was measured repeatedly
☐	☒	The statistical test(s) used AND whether they are one- or two-sided<br>*Only common tests should be described solely by name; describe more complex techniques in the Methods section.*
☒	☐	A description of all covariates tested
☐	☒	A description of any assumptions or corrections, such as tests of normality and adjustment for multiple comparisons
☐	☒	A full description of the statistical parameters including central tendency (e.g. means) or other basic estimates (e.g. regression coefficient) AND variation (e.g. standard deviation) or associated estimates of uncertainty (e.g. confidence intervals)
☐	☒	For null hypothesis testing, the test statistic (e.g. F, t, r) with confidence intervals, effect sizes, degrees of freedom and P value noted<br>*Give P values as exact values whenever suitable.*
☒	☐	For Bayesian analysis, information on the choice of priors and Markov chain Monte Carlo settings
☒	☐	For hierarchical and complex designs, identification of the appropriate level for tests and full reporting of outcomes
☐	☒	Estimates of effect sizes (e.g. Cohen's d, Pearson's r), indicating how they were calculated

Our web collection on statistics for biologists contains articles on many of the points above.

Software and code

Policy information about availability of computer code

Data collection: no software was used.

Data analysis	ChIP-seq :The prepared libraries were sequenced with 65 base single reads on Illumina Hiseq 2500. The sequencing reads were aligned to the mouse genome GRCm38 (mm10) using Burrows-Wheeler Aligner (BWA, v0.7.5a; (Li and Durbin, 2009) with a mapping quality >20. Peak calling was performed using both MACS2 v2.1.1.20160309 (q-value threshold 0.01, extension via Phantom Peaks) (Zhang et al., 2008). For each organoid and tumor dataset, the peaks from duplicate samples were merged based on the peak ranges using ChIPpeakAnno v3.18.2 (Zhu et al., 2010) and considered as MYC binding loci. The gene closest to each merged peak was defined as MYC target based on the GRCm38 (mm10) genome annotation.<br>RNAseq: The sequencing reads were aligned to the mouse genome GRCm38 (mm10) using TopHat v2.1 (Kim et al., 2013) and the number of reads mapped to each gene were quantified using HTSeq version 0.10.0 (Anders et al., 2015). DESeq2 v1.22.2 was used for read count normalization (median ratio method) and differential expression analysis. Genes with adjusted FDR<0.05 (Benjamini-Hochberg procedure) and |fold-changes|>1.5 were defined as differentially expressed genes.<br>R version 3.6.1 (2019-07-05)<br>http://genetica-network.com/(Urzúa-Traslaviña CG et al. Nat Commun. 2021; 12:1464.)<br>Graphpad Prism, version 8.4.2<br>ImageScope software version 12.0.0 (Aperio) or quPath version 0.3.0 (Bankhead et al., 2017) for analysis of immunohistochemistry slides.<br>ImageJ/Fiji version 1.53q was used to quantify splenocyte/organoid co-cultures<br>Leica Application Suite software version 4.13 was used to image human cell cultures<br>Zen software version 2.6 was used to image splenocyte/organoid co-cultures<br>Flowjo version 10 was used to analyze FACS data<br>Quantity One /Chemidoc XRS software version 4 (Bio-Rad) to analyze western blots

For manuscripts utilizing custom algorithms or software that are central to the research but not yet described in published literature, software must be made available to editors and reviewers. We strongly encourage code deposition in a community repository (e.g. GitHub). See the Nature Research guidelines for submitting code & software for further information.

Data

Policy information about availability of data

All manuscripts must include a data availability statement. This statement should provide the following information, where applicable:

- Accession codes, unique identifiers, or web links for publicly available datasets
- A list of figures that have associated raw data
- A description of any restrictions on data availability

Mouse RNA-seq and ChIP-seq data has been deposited with the ENA accession number “PRJEB43214” (https://www.ebi.ac.uk/ena/browser/view/PRJEB43214). The gene functionality analyzer tool is available at http://www.genetica-network.com. RNA-seq presented in Figure 1 has been deposited with GEO repository with identifier: GSE185512 (https://www.ncbi.nlm.nih.gov/geo/query/acc.cgi?acc=GSE185512)

Field-specific reporting

Please select the one below that is the best fit for your research. If you are not sure, read the appropriate sections before making your selection.

☒ Life sciences ☐ Behavioural & social sciences ☐ Ecological, evolutionary & environmental sciences

For a reference copy of the document with all sections, see nature.com/documents/nr-reporting-summary-flat.pdf

Life sciences study design

All studies must disclose on these points even when the disclosure is negative.

Sample size	For mouse studies, sample sizes were determined using power calculations, a power between 0.8 and 0.9 was deemed appropriate.
Data exclusions	No data was excluded, except if mice died before treatment was started in the tumor cohorts (deaths were not related to tumor growth in all cases).
Replication	All experiments were independently replicated 3 times (for cell line work), each mouse was treated as independent replicate
Randomization	Mice were allocated to treatment arms randomly. for in vitro experiments, no randomization was done, since there were no differences expected between different plates/wells.
Blinding	The technicians measuring tumor sizes were blinded to the treatment arms as were the pathologists and researchers assessing them.

Reporting for specific materials, systems and methods

We require information from authors about some types of materials, experimental systems and methods used in many studies. Here, indicate whether each material, system or method listed is relevant to your study. If you are not sure if a list item applies to your research, read the appropriate section before selecting a response.

Materials & experimental systems

n/a	Involved in the study
☐	☒ Antibodies
☐	☒ Eukaryotic cell lines
☒	☐ Palaeontology and archaeology
☐	☒ Animals and other organisms
☐	☒ Human research participants
☒	☐ Clinical data
☒	☐ Dual use research of concern

Methods

n/a	Involved in the study
☐	☒ ChIP-seq
☐	☒ Flow cytometry
☒	☐ MRI-based neuroimaging

Antibodies

Antibodies used

RAD51 (1:400, GeneTex, #gtx70230), Geminin (Cell Signaling, #9718, 1:200), cGAS (1:200, Cell Signaling, #15102), CD45-eFluor605NC (1:100; clone 30-F11, eBiosciences), CD11b-eFluor650NC (1:400; clone M1/70, eBiosciences), Ly6G-AlexaFluor700 (1:200; clone 1A8; BD Pharmingen), Ly6C-eFluor450 (1:400; clone HK1.4, eBiosciences), F4/80-PE (1:200; clone BM8, eBiosciences), CD49d-FITC (1:400; clone R1–2, eBiosciences), CD3 PerCP Cy5.5, CD206-FITC (1:200; clone C068C2, eBiosciences), 7-AAD (biolegend, cat. 420403); CD3-PE-Cy7 (1:200; clone 145–2C11, eBiosciences), CD4-APC-eFluor450 (1:200; clone GK1.5, eBioscience), CD8-PerCP-eFluor710 (1:400; clone 53–6.7, eBiosciences, CD49b-APC (1:400; clone DX5, eBiosciences), CD19-eFluor780 (1:200; clone eBio1D3), BRCA2 (1:1000, Calbiochem, #OP95), BRCA1 (1:1000, Cell Signaling, #9010), cGAS (1:1000, Cell Signaling, #15102), STING (1:1000, Cell Signaling, #13647), cMYC (1:200, Santa Cruz, sc40, Abcam (ab32072) 1:1000), pIRF3 (1:1000, Cell Signaling, # 29047), IRF3 (1:1000, Cell Signaling, # 4302), STAT1 (1:1000, Cell Signaling, # 9172), pSTAT1 (1:1000, Cell Signaling, # 8826), beta-Actin (1:10.000, MP Biochemicals, #69100).

Validation

eBiosciences guarantees specificity of the antibodies by expression level tests and/or confirmation of band size in western blots. Antibodies used for immuno-histochemistry are validated before use by the mouse pathology facility of the NKI by staining tissue with expression of the antigen versus antigen negative tissue. Cell signaling antibodies are guaranteed to be validated by the vendor. RAD51 is validated for IF by the vendor. For western blots, bands corresponded to expected height, and to loss of signal upon depletion or knock out. The cMYC antibody by SantaCruz was validated in house using tumors with and without MYC overexpression.

Eukaryotic cell lines

Policy information about cell lines

Cell line source(s)

all mouse cell lines: derived from mouse tumors grown at the NKI. Human cell lines were obtained form ATCC. Human breast cancer cell lines MDA-MB-231, HCC1806, BT-549 and HCC38 were obtained from ATCC (CRM-HTB-26, CRL-2335, HTB-122, CRL-2314).

Authentication

Mouse cell lines were authenticated via PCR test for the correct Cre-loxP mediated recombination events. STR profiling was used to authenticate the human cell line models.

Mycoplasma contamination

no mycoplasma was detected by PCR in the used cells

Commonly misidentified lines
(See ICLAC register)

No commonly misidentified lines were used in this study

Animals and other organisms

Policy information about studies involving animals; ARRIVE guidelines recommended for reporting animal research

Laboratory animals

female FVB mice, WapCre;Trp53F/F (WP), WapCre;Brca1F/F;Trp53F/F (WB1P), WapCre;Trp53F/F;Col1a1invCAG-Myc-IRES-Luc/+ (WP-Myc), WapCre;Brca1F/F;Trp53F/F;Col1a1invCAG-Myc-IRES-Luc/+ (WB1P-Myc), WapCre;Brca1F/F;Trp53F/F;Col1a1invCAG-Met-IRES-Luc/+ (WB1P-Met), and WapCre;Brca1F/F;Trp53F/F;Col1a1invCAG-Cas9-IRES-Luc/+ (WB1P-Cas9), WapCre;Brca1F/F;Trp53F/F;Col1a1invCAG-MycERT2-IRES-Luc/+ (WB1P-MycERT2) mice from an age of 7 weeks onwards were used in this study. Mice were housed with a 12/12 hour light/dark cycle, the humidity was between 45 and 65% and the temperature between 21 and 23 degrees celsius.

Wild animals

no wild animals were used in this study

Field-collected samples

no field collected samples were used in this study

Ethics oversight

All animal experiments were approved by the Animal Ethics Committee of the Netherlands Cancer Institute (Amsterdam, the Netherlands) and performed in accordance with the Dutch Act on Animal Experimentation.

Note that full information on the approval of the study protocol must also be provided in the manuscript.

Human research participants

Policy information about studies involving human research participants

Population characteristics	Random population of healthy volunteers (blood donors) from the Netherlands
Recruitment	Peripheral blood mononuclear cells (PBMCs) were isolated from randomly selected buffy coats obtained from healthy volunteers (Sanquin, Amsterdam. The Netherlands)
Ethics oversight	The procedure was approved by the Ethical Advisory Council located in Amsterdam, The Netherlands. All experiments using human PBMCs were in compliance with the Helsinki declaration and written informed consent was obtained from all donors

Note that full information on the approval of the study protocol must also be provided in the manuscript.

ChIP-seq

Data deposition

☒ Confirm that both raw and final processed data have been deposited in a public database such as GEO.

☒ Confirm that you have deposited or provided access to graph files (e.g. BED files) for the called peaks.

Data access links<br>*May remain private before publication.*	European Nucleotide Archive - PRJEB43214<br>https://www.ebi.ac.uk/ena/browser/view/PRJEB43214
Files in database submission	5395_3_wz3427_AACAACCA_S85_R1_001.fastq.gz<br>5395_4_wz3428_AACCGAGA_S89_R1_001.fastq.gz<br>5395_5_wz3429_ACGCTCGA_S90_R1_001.fastq.gz<br>5395_6_wz3430_ACGTATCA_S88_R1_001.fastq.gz<br>5634_5_wz3701_CGACGTTA_S52_R1_001.fastq.gz<br>5634_6_wz3702_TAACCGGT_S53_R1_001.fastq.gz<br>5634_9_wz3705_TGTACCGT_S56_R1_001.fastq.gz<br>5634_10_wz3706_TGACTGAC_S57_R1_001.fastq.gz<br>5395_3_wz3427_AACAACCA_S85.filtered.bam<br>5395_4_wz3428_AACCGAGA_S89.filtered.bam<br>5395_5_wz3429_ACGCTCGA_S90.filtered.bam<br>5395_6_wz3430_ACGTATCA_S88.filtered.bam<br>5634_5_wz3701_CGACGTTA_S52.filtered.bam<br>5634_6_wz3702_TAACCGGT_S53.filtered.bam<br>5634_9_wz3705_TGTACCGT_S56.filtered.bam<br>5634_10_wz3706_TGACTGAC_S57.filtered.bam
Genome browser session<br>(e.g. UCSC)	no longer applicable

Methodology

Replicates	two replicates for tumors and organoids, 5 replicates for Myc and Miz1
Sequencing depth	65bp read length, single-end sequencing
Antibodies	cMYC antibody Y69 (Abcam), Miz1 10E2
Peak calling parameters	MACS2 v2.1.1.20160309 (q-value threshold 0.01, extension via Phantom Peaks)
Data quality	Burrows-Wheeler Aligner (BWA, v0.7.5a) with a mapping quality >20<br>MACS2 v2.1.1.20160309 (q-value threshold 0.01, extension via Phantom Peaks)
Software	Burrows-Wheeler Aligner (BWA, v0.7.5a)<br>MACS2 v2.1.1.20160309<br>ChIPpeakAnno v3.18.2

Flow Cytometry

Plots

Confirm that:

☒ The axis labels state the marker and fluorochrome used (e.g. CD4-FITC).

☒ The axis scales are clearly visible. Include numbers along axes only for bottom left plot of group (a 'group' is an analysis of identical markers).

☒ All plots are contour plots with outliers or pseudocolor plots.

☒ A numerical value for number of cells or percentage (with statistics) is provided.

Methodology

Sample preparation	Mouse tissues were collected in ice-cold PBS. Blood samples were collected in tubes containing heparin (Leo Pharma) and treated with red blood cell lysis buffer (155mM NH4CL, 12mM NaHCO3, 0.1mM EDTA) (RBC). Tumors were mechanically chopped using a McIlwain Tissue Chopper (Mickle Laboratory Engineering) and digested either for 1 hour at 37°C in a digestion mix of 3 mg/ml collagenase type A (Roche, 11088793001) and 25 µg/ml DNAse (Invitrogen, 18068–015) or for 30 min at 37°C in 100 µg/ml Liberase (Roche, 5401127001), in serum-free DMEM (Invitrogen). Reactions were terminated by addition of DMEM containing 8% FCS and cell suspensions were dispersed through a 70 µm cell strainer (BD Falcon, 352350). All single-cell suspensions were treated with RBC lysis buffer to remove red blood cells. Single-cell suspensions were plated in equal numbers in round bottom 96-wells plates (Thermo Scientific). Cells were incubated with mouse Fc BlockTM (BD Biosciences) for 15 min at 4°C and subsequently incubated with different combinations of fluorescently labeled monoclonal antibodies for 20 min in the dark at 4°C<br><br>Human cell lines Cells were washed with 1% BSA-PBS and permeabilized with Perm Buffer III (BD bioscience) for 30 min. on ice. Samples were washed with 1% BSA-PBS and incubated (150.000 cells per sample) with pIRF3 primary antibody (1:100, Cell signaling, #29047, clone D601M) for 1 hour at 4 °C and subsequently stained with AlexaFluor 488-conjugated goat anti-rabbit secondary antibody (1:300) for 1 hour at RT.
Instrument	BD LSRII using Diva Software (BD Biosciences) and FACS Calibur (Becton Dickinson)
Software	FlowJo Software version 10.0 (Tree Star Inc.)
Cell population abundance	After sorting for ECAD+ cells to perform RNA seq (Figure 3a), a fraction of cells sorted as Ecad+ was analyzed by FACS to check purity. Purity reached was 59%, 10% was CD45+ contamination, the rest was debris respectively only relatively weakly positive.
Gating strategy	Gating was performed by using FSC-A/SSC-A, then an FSC-A/FSC-H gate to define single cells (doublets were assumed to drop below the straight line in FSC-A vs H). Live/dead stain was used to exclude dead cells. Contour plots were used to distinguish between populations more clearly. All cells negative for live/dead stain were used for further analysis by the respective panels.

☒ Tick this box to confirm that a figure exemplifying the gating strategy is provided in the Supplementary Information.
